# For patients with prior coronary artery bypass grafting and recurrent myocardial ischemia, percutaneous coronary intervention on bypass graft or native coronary artery?—A 5‐year follow‐up cohort study

**DOI:** 10.1002/clc.24021

**Published:** 2023-04-28

**Authors:** Ru Liu, Haibo Liu, Deshan Yuan, Yan Chen, Xiaofang Tang, Ce Zhang, Pei Zhu, Tao Yang, Yongbao Zhang, Han Li, Ou Xu, Runlin Gao, Bo Xu, Jinqing Yuan

**Affiliations:** ^1^ Department of Cardiology, Fuwai Hospital Chinese Academy of Medical Sciences Beijing China; ^2^ Department of Respiratory and Pulmonary Vascular Disease Fuwai Yunnan Cardiovascular Hospital Kunming China; ^3^ Department of Adult Cardiac Surgery, Fuwai Hospital Chinese Academy of Medical Sciences Beijing China; ^4^ Department of Vascular Surgery, Fuwai Hospital Chinese Academy of Medical Sciences Beijing China

**Keywords:** coronary artery bypass grafting, coronary artery disease, percutaneous coronary intervention

## Abstract

**Background:**

Real‐world data on target vessel of percutaneous coronary intervention (PCI) for patients with prior coronary artery bypass grafting (CABG) was still limited.

**Hypothesis:**

A prospective cohort was examined to determine the frequency and outcomes of native coronary artery PCI versus bypass graft PCI in patients with prior CABG.

**Methods:**

A large‐sample observational study enrolled a total of 10 724 patients with coronary artery disease (CAD) underwent PCI in 2013. Two‐ and five‐year clinical outcomes were compared between graft PCI group and native artery PCI group in patients with prior CABG.

**Results:**

A total of 438 cases had CABG history in the total cohort. Graft PCI group and native artery PCI group accounted for 13.7% and 86.3%, respectively. The rates of 2‐ and 5‐year all‐cause death and major adverse cardiovascular and cerebral events (MACCE) showed no significant difference between the two groups (*p* > .05). Two‐year revascularization risk was lower in graft PCI group than native artery PCI group (3.3% and 12.4%, *p* < .05), but 5‐year myocardial infarction (MI) risk was higher (13.3% and 5.0%, *p* < .05). In multivariate COX regression models, graft PCI group was independently associated with lower 2‐year revascularization risk (hazard ratio [HR]: 0.21; 95% confidence interval [CI]: 0.05–0.88; *p* = .033), but higher 5‐year MI risk than native artery PCI group (HR: 2.61; 95% CI: 1.03–6.57; *p* = .042). Five‐year all‐cause death and MACCE risk showed no difference between the two groups in model.

**Conclusions:**

In patients with prior CABG underwent PCI, patients in graft PCI group had higher 5‐year MI risk than patients received native artery PCI. But, 5‐year mortality and MACCE was not significantly different between graft PCI group and native artery PCI group.

## Graphical abstract

This study examined a large prospective cohort to determine the frequency and outcomes of native coronary artery percutaneous coronary intervention (PCI) versus bypass graft PCI in patients with prior coronary artery bypass graft (CABG) and recurrent myocardial ischemia. A total of 438 cases had prior CABG history in the total cohort enrolled 10 724 cases. Graft PCI group and native artery PCI group accounted for 13.7% and 86.3%, respectively. The rates of 2‐ and 5‐year all‐cause death and major adverse cardiovascular and cerebral events (MACCE) showed no significant difference between the two groups. Two‐year revascularization risk was lower in graft PCI group than native artery PCI group, but 5‐year myocardial infarction risk was higher. In multivariate COX regression models, compared with native artery PCI group, graft PCI group were independently associated with decreased 2‐year revascularization risk, but higher 5‐year myocardial infarction risk. But, the 5‐year all‐cause death and MACCE risk were similar between graft PCI group and native artery PCI group.

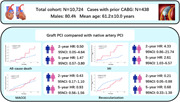

## 1 INTRODUCTION

Coronary artery bypass grafting (CABG) has been applicated for more than 50 years in patients with severe coronary artery disease (CAD).^1^ In patients with prior CABG, atherosclerosis might progress, with newly occurred stenosis or occlusion in the graft.^2^, ^3^, ^4^ And patients with chronic bypass graft lesions often present with recurrent angina pectoris, myocardial infarction (MI), even sudden death. The strategy and technique of revascularization for this special population remained a difficult problem in the interventional treatment area of CAD. Simple optimization of drug therapy often cannot control myocardial ischemia in such patients. However, secondary thoracotomy for re‐CABG is supposed not the first choice due to anatomical changes, tissue adhesions, and the source of bridging vessels. Therefore, percutaneous coronary intervention (PCI) has become the first choice of treatment strategy for recurrent myocardial ischemia after CABG.^5^, ^6^, ^7^, ^8^

It is widely believed that native coronary arteries should be the preferred target vessel of PCI in patients with prior CABG, if technically feasible, because native coronary artery PCI appears to be associated with better short‐ and long‐term outcomes compared with bypass graft PCI.^9^, ^10^ However, patients with prior CABG and recurrent myocardial ischemia tend to be elderly, with severe myocardial ischemia, poor cardiac function, and many comorbidities, there is a lack of randomized controlled trials (RCT) or large sample retrospective data for comparison of efficacy and safety between native artery intervention and graft intervention.^11^, ^12^, ^13^ Clinical practice still emphasizes the principle of individualization. Moreover, with widespread use of the second‐generation drug‐eluting stents and development of drug therapy, including stronger P2Y12 receptor antagonists and anti‐reflow medication, the risk of graft intervention has been reduced.^14^ In the present situation, how to select target vessels in patients with prior CABG also requires new data.

Therefore, we examined a large‐sample all‐comer observational cohort, to compare the outcomes of patients with CABG history who received PCI on native arteries and graft arteries due to recurrent myocardial ischemia, aimed to provide some real‐world data for optimization of target vessel selection in patients with prior CABG.

## 2 PATIENTS AND METHODS

### 2.1 Ethical statement

Ethical approvals were obtained from the Fuwai Hospital Research Ethics Committees (No. 2020‐1310). The Institutional Review Board approved the study protocol and all patients signed written informed consent before the intervention, including full set of risk‐informed consent and information use consent for scientific purposes. By the time we formed the database, we have deidentified all patient details.

### 2.2 Study population

A total of 10 724 consecutive cases with CAD who underwent PCI were included from January to December 2013 in Fuwai Hospital, the largest cardiovascular center in China. Diagnosis of ST‐segment elevated myocardial infarction, non‐ST‐segment elevated myocardial infarction, unstable angina pectoris, and stable angina pectoris was in terms of criteria based on the international guidelines.^15^, ^16^, ^17^

### 2.3 Procedural details

Before elective PCI, if not taking long‐term aspirin and clopidogrel, patients received aspirin and P2Y12 inhibitor with loading dose orally. Patients with acute coronary syndrome scheduled for primary PCI received the same dose of aspirin and clopidogrel (loading dose 300 or 600 mg, according to bleeding risk) as soon as possible. Before coronary angiography (CAG), 25 mg heparin sodium was administered through an arterial sheath or intravenously. Before PCI, 100 U/kg of heparin sodium was administered. The dose was lowered to 50–70 U/kg in patients over the age of 70 to reduce bleeding risk. If PCI proceeded for more than 1 h, an additional 1000 U of heparin sodium was administered. Results of CAG were read by experienced cardiologists. More than 50% stenosis of left main artery (LM), left anterior descending artery (LAD), left circumflex artery (LCX), right coronary artery (RCA), and main branch of these vessels was defined as coronary artery stenosis. More than 70% stenosis of the vessels mentioned above, along with ischemic symptoms or ischemic evidence showed by examinations, was indicated for coronary stent implantation. Three‐vessel disease (TVD) was defined as angiographic stenosis of ≥50% in all three main coronary arteries, LAD, LCX, and RCA.

### 2.4 Follow‐up and definitions

The patients were visited 30 days and 6 months after PCI and every 1 year thereafter. Information of in‐hospital outcome was obtained through review of medical records, and the long‐term clinical outcome was collected from survey completed by telephone follow‐up. A group of independent clinical physicians oversaw checking and confirmed all adverse events carefully. Investigators training, blinded questionnaire filling, and telephone recording were performed to control the data quality.

Primary endpoint was all‐cause death. Composite endpoint was defined as major adverse cardiovascular and cerebrovascular events (MACCE), including all‐cause death, revascularization, MI, and stroke. Secondary endpoints were MACCE, cardiac death, revascularization, MI, stroke, and bleeding. Cardiac death is identified as death caused by MI, heart failure, and/or malignant arrhythmia definitely; or death which cannot be explained clearly by other reasons. Stent thrombosis (ST) was defined on the basis of Academic Research Consortium definitions according to the level of certainty as definite and probable.^18^ Bleeding was defined according to criteria established by Bleeding Academic Research Consortium (BARC), excluding BARC 0 and 1 type.^19^

### 2.5 Statistical analysis

Data statistics was applied using SPSS 22.0 (IBM Corp.). Student's *t*‐tests were used to compare the normally distributed continuous variables between the two groups. Chi‐square tests were applied to compare categorical variables between the two groups. Kaplan–Meier curves were drawn to compare cumulative event rates of the two groups. Multivariate COX proportional hazard regression analyses were applied to control baseline confounders. Covariates for COX regression were those variables with significant differences in baseline or important clinical meaning. All *p*‐values were two‐sided with a significance level of <.05.

## 3 RESULTS

### 3.1 Baseline characteristics

Among the total cohort, there were 438 cases with prior CABG. There were 60 patients who received graft artery PCI and 378 cases received native artery PCI, accounted for 13.7% and 86.3%, respectively. In graft PCI group, only five patients received left internal mammary artery (LIMA) PCI, and other 55 cases received saphenous vein graft PCI. Patients in graft PCI group were presented with more male gender, older age, more previous MI, more family history of CAD, lower left ventricular ejection fraction, less LM or TVD, less LAD involved, less trans radial approach and pulling out sheath directly, less intravascular ultrasound application and shorter time of procedure compared with native artery PCI group (all *p* < .05) (Table 1).

**Table 1:** The baseline clinical characteristics.

Variables	Patients with prior CABG	Graft PCI	Native artery PCI	*p*‐value
	(*n* = 438)	(*n* = 60)	(*n* = 378)	
Demographic characteristics				
Male gender (%)	352 (80.4)	55 (91.7)	297 (78.6)	.018
Age (years)	61.2 ± 10.0	64.3 ± 10.1	60.7 ± 9.9	.01
BMI (kg/m^2^)	26.1 ± 3.1	25.9 ± 2.6	26.1 ± 3.2	.643
Coexisting conditions (%)				
Hypertension	300 (68.5)	42 (70.0)	258 (68.3)	.787
DM	156 (35.6)	22 (36.7)	134 (35.4)	.855
Hyperlipidemia	324 (74.0)	43 (71.7)	281 (74.3)	.661
Previous MI	137 (31.3)	31 (51.7)	106 (28.0)	<.001
Current smoker	174 (39.7)	22 (36.7)	152 (40.2)	.602
Family history of CAD	102 (23.3)	21 (35.0)	81 (21.4)	.021
CVD	31 (7.1)	5 (8.3)	26 (6.9)	.683
PVD	22 (5.0)	5 (8.3)	17 (4.5)	.206
COPD	11 (2.5)	2 (3.3)	9 (2.4)	.661
LVEF (%)	60.7 ± 8.6	58.4 ± 8.0	61.1 ± 8.7	.024
Clinical presentation (%)				.565
Asymptomatic ischemia	23 (5.3)	1 (1.7)	22 (5.8)	
Stable angina	178 (40.6)	27 (45.0)	151 (39.9)	
Unstable angina pectoris	200 (45.7)	27 (45.0)	173 (45.8)	
AMI	37 (8.4)	5 (8.3)	32 (8.5)	.973
Laboratory examination				
eGFR before PCI (mL/min/1.73 m^2^)	86.4 ± 16.3	85.6 ± 13.7	86.5 ± 16.7	.717
HGB before PCI (g/L)	137.9 ± 16.1	139.2 ± 13.9	137.7 ± 16.5	.513
PLT before PCI (10^9^/L)	190.6 ± 51.3	180.0 ± 50.3	192.3 ± 51.3	.085
Uric acid (µmol/L)	348.8 ± 91.5	336.8 ± 85.5	350.7 ± 92.4	.277
HbA1c (%)	6.8 ± 1.3	6.9 ± 1.3	6.8 ± 1.3	.463
LDL‐C (mmol/L)	2.4 ± 0.9	2.5 ± 1.0	2.4 ± 0.9	.316
ESR (mm/h)	10.5 ± 10.8	9.2 ± 7.3	10.7 ± 11.2	.322
Angiographic and procedural characteristics				
LM or TVD (%)	33 (7.5)	0 (0.0)	33 (8.7)	.017
LAD involved (%)	383 (87.4)	45 (75.0)	338 (89.4)	.002
No. of target lesions	1.4 ± 0.7	1.4 ± 0.7	1.4 ± 0.7	.86
No. of stent per patient	1.8 ± 1.2	1.8 ± 1.1	1.9 ± 1.2	.494
Trans radial approach	237 (54.1)	18 (30.0)	219 (57.9)	<.001
Pulling out sheath directly	234 (53.4)	16 (26.7)	218 (57.7)	<.001
IVUS application	43 (9.8)	0 (0.0)	43 (11.4)	.006
Time of procedure, min)	46.7 ± 43.9	34.8 ± 23.9	48.6 ± 46.0	.001
Procedure and stent type (%)				.079
PTCA	13 (3.0)	2 (3.3)	11 (2.9)	
BMS	6 (1.4)	2 (3.3)	4 (1.1)	
First‐generation durable polymer DES	25 (5.7)	7 (11.7)	18 (4.8)	
Second‐generation durable polymer DES	246 (56.2)	29 (48.3)	217 (57.4)	
Domestic biodegradable polymer DES	46 (10.5)	2 (3.3)	44 (11.6)	
Mixed implantation of DES	80 (18.3)	15 (25.0)	65 (17.2)	
Others (Janus, Yinyi)	3 (0.7)	0 (0.0)	3 (0.8)	
Procedure unsuccess	19 (4.3)	3 (5.0)	16 (4.2)	
Medication (%)				
Aspirin	431 (98.4)	59 (98.3)	372 (98.4)	.964
Clopidogrel	438 (100.0)	60 (100.0)	378 (100.0)	
Statin	422 (96.3)	57 (95.0)	365 (96.6)	.549
Calcium antagonist	235 (53.7)	38 (63.3)	197 (52.1)	.106
β‐blocker	413 (94.3)	58 (96.7)	355 (93.9)	.393

*Note*: Data are expressed as mean ± standard deviation; or counts (percentage). BMI was defined as weight in kilograms divided by height in meters squared (kg/m^2^), using the Cooperative Meta‐analysis Group of China Obesity Task Force BMI classification.

Abbreviations: AMI, acute myocardial infarction; BMI, body mass index; BMS, bare metal stent; CAD, coronary artery disease; CABG, coronary artery bypass grafting; COPD, chronic obstructive pulmonary disease; CVD, cerebral vascular disease; DES, drug‐eluting stent; DM, diabetes mellitus; eGFR, estimated glomerular filtration rate; ESR, erythrocyte sedimentation rate; HbA1c, hemoglobin A1c; HGB, hemoglobin; IVUS, intravascular ultrasound; LAD, left anterior descending artery; LM, left main; LDL‐C, low density lipoprotein cholesterol; LVEF, left ventricular ejection fraction; MI, myocardial infarction; PCI, percutaneous coronary intervention; PLT, platelet; PTCA, percutaneous transluminal coronary angioplasty; PVD, peripheral vascular disease; TVD, three‐vessel disease.

There were 591 lesions among 438 cases with prior CABG. Patients in graft PCI group presented with less bifurcation lesions and more thrombotic lesions (*p* < .001), compared with native artery PCI group. There were 87.4% patients with LAD involved. In all target lesions, B2 and C lesions accounted for 77.3%. Moderate or severe calcification accounted for 19.6%, chronic total occlusion (CTO) accounted for 24%, Ostial lesions accounted for 23.2%. Bifurcation lesions were more while thrombotic lesions were less in native artery PCI group than graft PCI group (Tables 1 and 2).

**Table 2:** The baseline lesion characteristics.

	Patients with prior CABG	Graft PCI	Native artery PCI	*p*‐value
	(*n* = 591)	(*n* = 67)	(*n* = 524)	
TIMI flow before PCI				.031
0	141 (23.9)	12 (17.9)	129 (24.6)	
1	10 (1.7)	2 (3.0)	8 (1.5)	
2	59 (10.0)	13 (19.4)	46 (8.8)	
3	381 (64.5)	40 (59.7)	341 (65.1)	
TIMI flow after PCI				.047
0	22 (3.7)	2 (3.0)	20 (3.8)	
1	1 (0.2)	1 (1.5)	0 (0.0)	
2	9 (1.5)	1 (1.5)	8 (1.5)	
3	559 (94.6)	63 (94.0)	496 (94.7)	
De novo	552 (93.4)	60 (89.6)	492 (93.9)	.034
B2 or C lesions	457 (77.3)	55 (82.1)	402 (76.7)	.323
Moderate or severe calcification	116 (19.6)	14 (20.9)	102 (19.5)	.781
CTO lesions	142 (24.0)	13 (19.4)	129 (24.6)	.347
Ostial lesions	137 (23.2)	14 (20.9)	123 (23.5)	.638
Bifurcation lesions	98 (16.6)	0 (0.0)	98 (18.7)	<.001
Thrombotic lesions	21 (3.6)	10 (14.9)	11 (2.1)	<.001

Abbreviations: CABG, coronary artery bypass grafting; CTO, chronic total occlusion; PCI, percutaneous coronary intervention; TIMI, thrombolysis in myocardial infarction.

### 3.2 2‐ and 5‐year clinical outcomes

**Figure 1:**
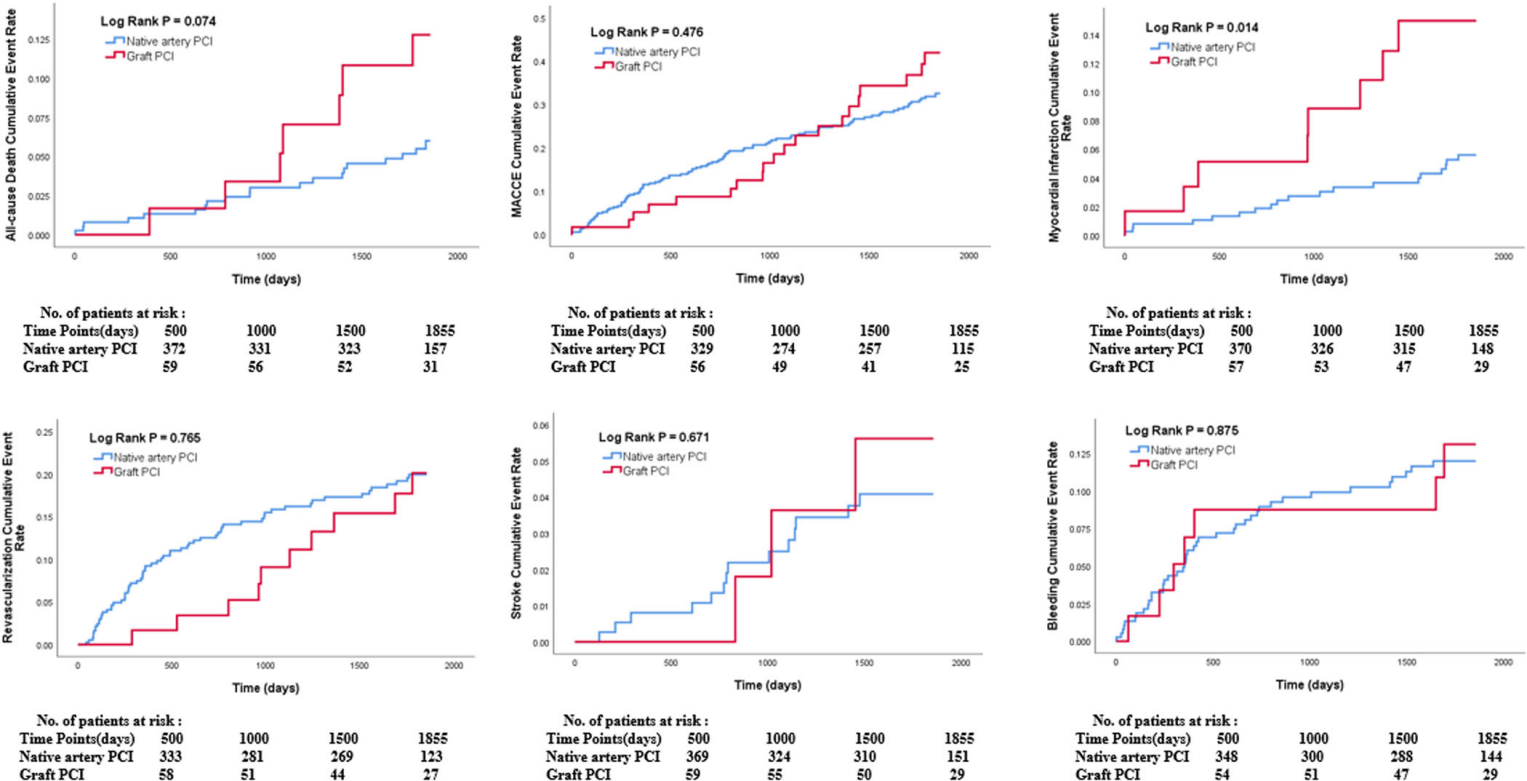
5‐year Kaplan–Meier survival curves.

For the analyzed population, clinical follow‐up was completed for all patients. The occurrence of adverse cardiovascular and cerebrovascular events in each group is listed in Table 3. The rates of 2‐ and 5‐year all‐cause death and MACCE were all not significantly different among the two groups. The rate of 2‐year revascularization showed significantly lower in graft PCI group than native artery PCI group (3.3% and 12.4%, *p* = .038). Although the rate of 2‐year stent thrombosis was higher in graft PCI group than native artery PCI group (5.0% and 1.1%, *p* = .024), but 2‐year MI rate showed no difference between the two groups (5.0% and 1.9%, *p* = .129). Extended follow‐up time, the rate of 5‐year MI presented significancy between the two groups. Patients in graft PCI group showed higher 5‐year MI risk than patients in native artery PCI group (13.3% and 5.0%, *p* = .013). Kaplan–Meier curves revealed the same results (Figure 1).

**Table 3:** 2‐ and 5‐year outcomes.

2‐year outcomes					5‐year outcomes			
Endpoints	Patients with prior CABG	Graft PCI	Native artery PCI	*p*‐value	Patients with prior CABG	Graft PCI	Native artery PCI	*p*‐value
	(*n* = 438)	(*n* = 60)	(*n* = 378)		(*n* = 438)	(*n* = 60)	(*n* = 378)	
All‐cause death	9 (2.1)	1 (1.7)	8 (2.1)	.82	27 (6.2)	7 (11.7)	20 (5.3)	.056
MACCE	66 (15.1)	5 (8.3)	61 (16.1)	.116	120 (27.4)	20 (33.3)	100 (26.5)	.267
Cardiac death	8 (1.8)	1 (1.7)	7 (1.9)	.921	18 (4.1)	5 (8.3)	13 (3.4)	.076
Myocardial infarction	10 (2.3)	3 (5.0)	7 (1.9)	.129	27 (6.2)	8 (13.3)	19 (5.0)	.013
Stent thrombosis	7 (1.6)	3 (5.0)	4 (1.1)	.024	7 (1.6)	3 (5.1)	4 (1.1)	.022
Revascularization	49 (11.2)	2 (3.3)	47 (12.4)	.038	75 (17.1)	10 (16.7)	65 (17.2)	.919
Stroke	5 (1.1)	0 (0.0)	5 (1.3)	.37	17 (3.9)	3 (5.0)	14 (3.7)	.629
Bleeding	28 (6.4)	2 (3.3)	26 (6.9)	.297	48 (11.0)	7 (11.7)	41 (10.8)	.85

Abbreviations: CABG, coronary artery bypass grafting; MACCE, major adverse cardiovascular and cerebrovascular events; PCI, percutaneous coronary intervention.

Multivariable COX regression models were built, including possible confounders at baseline (*p* < .05) or those having important clinical meaning. Five‐year all‐cause death and MACCE risk showed no difference between the two groups in model. Compared with native artery PCI group, graft PCI group was independently associated with decreased 2‐year revascularization risk (hazard ratio [HR]: 0.21, 95% confidence interval [CI]: 0.05–0.88, *p* = .033), but higher 5‐year MI risk (HR: 2.61, 95% CI: 1.03–6.57, *p* = .042) (Table 4).

**Table 4:** Multivariate COX regression analysis of 2‐ and 5‐year outcomes.

Endpoints	2‐year		5‐year	
	HR (95% CI)	*p*‐value	HR (95% CI)	*p*‐value
All‐cause death	0.50 (0.05–4.64)	.543	1.47 (0.57–3.80)	.428
MACCE	0.43 (0.17–1.10)	.078	0.93 (0.56–1.56)	.784
Cardiac death	0.72 (0.07–7.10)	.782	1.82 (0.56–5.88)	.317
Myocardial infarction	4.33 (0.86–21.74)	.075	2.61 (1.03–6.57)	.042
Stent thrombosis	3.99 (0.71–22.46)	.117	4.05 (0.72–22.87)	.114
Revascularization	0.21 (0.05–0.88)	.033	0.68 (0.33–1.38)	.285
Stroke	—	—	1.11 (0.30–4.15)	.877
Bleeding	0.53 (0.12–2.35)	.401	1.01 (0.43–2.37)	.974

Abbreviations: CABG, coronary artery bypass grafting; CI, confidence interval; HR, hazard ratio; MACCE, major adverse cardiovascular and cerebrovascular events; PCI, percutaneous coronary intervention.

## 4 DISCUSSION

In patients with prior CABG, their native coronary arteries are probably not able to achieve complete revascularization by PCI, due to multi‐vessel lesions, diffuse severe stenosis coexisted calcification or CTO, and that is the reason they have to undergo CABG. When they suffer recurrent myocardial ischemia, most of them were recommended to optimize drug therapy first, mainly due to unfeasible re‐CABG or PCI technology. The technical challenges of secondary CABG surgery mainly include the origin of bypass graft, internal tissue adhesion, and anatomical structure changes.^12^, ^13^ It was reported that the incidence of death, MI, perioperative complications were significantly increased, and clinical benefit was decreased in the re‐CABG patients, compared with the first‐time CABG patients. Very few patients receive secondary CABG.^20^ However, when optimal medication cannot able to control the symptoms of myocardial ischemia well, patients are re‐admitted and resorted to revascularization. And as a compromise, they receive partial revascularization by PCI to achieve some improvement in quality of life, even cardiac function. The progress of PCI technology and equipment plays a key role. It was reported that patients undergoing redo CABG were more complex and associated with worse clinical outcomes than those receiving PCI.^21^ The absolute survival benefit of successful CTO procedures was more pronounced in patients with previous CABG than in non‐CABG patients.^22^ Therefore, PCI gradually become the first choice on the basis of “technical” feasibility in patients with prior CABG and recurrent myocardial ischemia.^23^

In this study, 87.4% of the patients had LAD lesions, and most of the lesions were type B2 or C. Only 7.5% of the patients had LM involved or TVD, lower than expected. Since the population we analyzed in this study was all patients with recurrent myocardial ischemia after CABG undergoing PCI, it is conceivable that they were patients who still had the opportunity for intervention. Patients who did not have the opportunity for intervention and chose to take medicine were not included in the analysis. Diabetes was present in 35.6% of patients with prior CABG in this study. These real‐world data reflected that treatment situation was reasonable and guideline‐followed. CABG was found superior to PCI in patients with multivessel disease and diabetes.^23^ CABG (with a LIMA to the LAD) is recommended in preference to PCI to reduce mortality and repeat revascularization, in patients with diabetes and multivessel CAD with the involvement of LAD, who are appropriate candidates for CABG.^24^

This study showed all‐cause death and MACCE risk was the same between patients who receive graft PCI and cases who received native artery PCI. This was not consistent with previous studies. For example, a data from the National Cardiovascular Data Registry CathPCI Registry showed that bypass graft PCI is independently associated with higher in‐hospital mortality compared with native coronary PCI.^9^ Another national cohort study of 11 118 veterans with prior CABG who underwent PCI showed: compared with native coronary PCI, bypass graft PCI was significantly associated with higher incidence of short‐ and long‐term major adverse events, including more than double the rate of in‐hospital mortality.^10^ This study suggested whether intervention in its native coronary arteries or bypass graft, patients' long‐term mortality had no obvious difference. There are several possible reasons: (1) the second‐generation durable polymer DES and domestic biodegradable polymer DES accounted for 66.7% in all cases analyzed in this study; (2) experience of perioperative management accumulated, especially the progress of drug treatment, including antithrombotic and microcirculation improving therapy, such as nitric acid ester, nicorandil and Chinese patent medicine as adjuvant therapy like Tongxinluo capsule,^25^ as well as application of intracoronary anti‐no‐reflow treatment during procedure.

Interestingly, results showed that patients in graft PCI group had lower 2‐year revascularization risk and higher 5‐year MI risk than native artery PCI group. Because this is an observational cohort study, we still cannot conclude that native artery PCI is superior to graft artery PCI. The decrease of revascularization within 2 years may be due to the high difficulty of self vascular intervention in the patients who choose to intervene in the graft, so there are fewer patients who have the opportunity to treat their native vessels within 2 years. In other words, the graft intervention was chosen possibly because native artery intervention could not be performed again. Therefore, the lower rate of revascularization events we saw within 2 years might not be due to the reduction of ischemic events following graft PCI, but due to the baseline bias. On the other hand, the increased risk of recurrent MI might also be due to the poor vascular condition and progression of atherosclerosis of patients in graft PCI group. It could be concluded that there is no difference in long‐term death between the two groups, PCI in graft or native artery are both feasible methods in patients with prior CABG and recurrent myocardial ischemia.

In addition to baseline bias described above, there were several limitations in this study. First, ticagrelor was seldom used in our center in the year 2013. It was prescribed only when “clopidogrel resistance” was observed and patients were willing to take it on their own expense. All patients included in the analysis remained on anti‐thrombotic therapy with aspirin and clopidogrel, which may have contributed to stent thrombosis and recurrent MI to some extent. Second, the application of endovascular imaging, optical coherence tomography and intravascular ultrasound, was relatively low in this cohort study in 2013. Nevertheless, this is a core laboratory analysis comparing the efficacy and safety between bypass graft PCI and native coronary artery PCI in patients with prior CABG and recurrent myocardial ischemia, in terms of both long‐term outcomes and angiographic data, and we believe that we have accounted for the most clinically relevant variables in our model.

## 5 CONCLUSIONS

In patients with prior CABG and recurrent myocardial ischemia undergoing PCI, compared with patients who received native artery PCI, those who received graft PCI had lower 2‐year revascularization risk, but higher 5‐year MI risk. However, 5‐year all‐cause death and MACCE were similar between graft PCI group and native artery PCI group.

## AUTHOR CONTRIBUTIONS

Ru Liu contributed to all aspects of this study, including study concept and design, data acquisition, statistical analysis and interpretation, drafting and revising the report, and funding. Haibo Liu, Deshan Yuan, Yan Chen, Xiaofang Tang, Ce Zhang, Pei Zhu, Yongbao Zhang, Tao Yang, Han Li, Ou Xu, and Runlin Gao contributed to data acquisition and ethical issues. Bo Xu and Jinqing Yuan contributed to initial study conception and design, and funding. All authors have approved the final article.

## CONFLICT OF INTEREST STATEMENT

The authors declare no conflict of interest.

## Data Availability

The data used to support the findings of this study are available from the corresponding author upon request.
